# Supplementary material for: Legacy copper/nickel mine tailings potentially harbor novel iron/sulfur cycling microorganisms within highly variable communities
Source: Appl Environ Microbiol. 2024 May 30;90(6):e00143-24. doi: 10.1128/aem.00143-24 (PMC11218620; doi:10.1128/aem.00143-24)
Supplement: Supplemental material — Figures S1 to S6; Table S1. [file aem.00143-24-s0002.pdf]

Legacy copper/nickel mine tailings potentially harbor novel iron/sulfur cycling microorganisms within highly variable communities

Running title: Novel mineral cycling microbes in mine tailings

Chen, Molly[a]; Grégoire, Daniel S. [a,b]; Bain, Jeffrey G. [c]; Blowes, David W. [c]; Hug, Laura A. [a,#]

a – Department of Biology, University of Waterloo, Waterloo, Ontario, Canada
b – Department of Chemistry, Carleton University, Ottawa, Ontario, Canada
c – Department of Earth and Environmental Sciences, University of Waterloo, Waterloo, Ontario, Canada
# - corresponding author, laura.hug@uwaterloo.ca

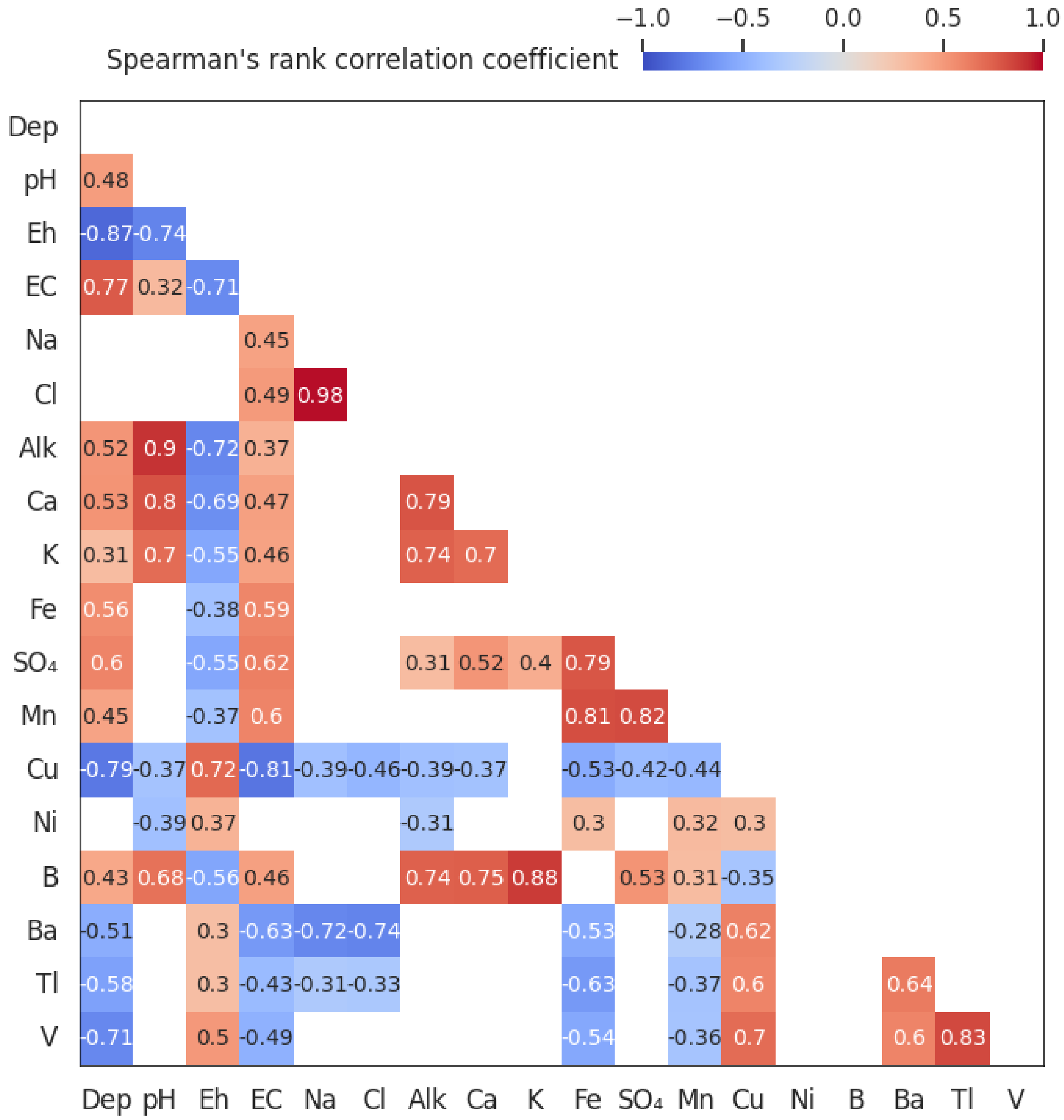

Supplemental Figure 1: Correlation matrix of geochemical variables. Pairwise Spearman's rank correlation coefficients were calculated for each factor. Dep = depth, EC = electrical conductivity, Alk = Alkalinity. Significant correlations ($p<0.05$, Bonferroni corrected) are displayed in the figure.

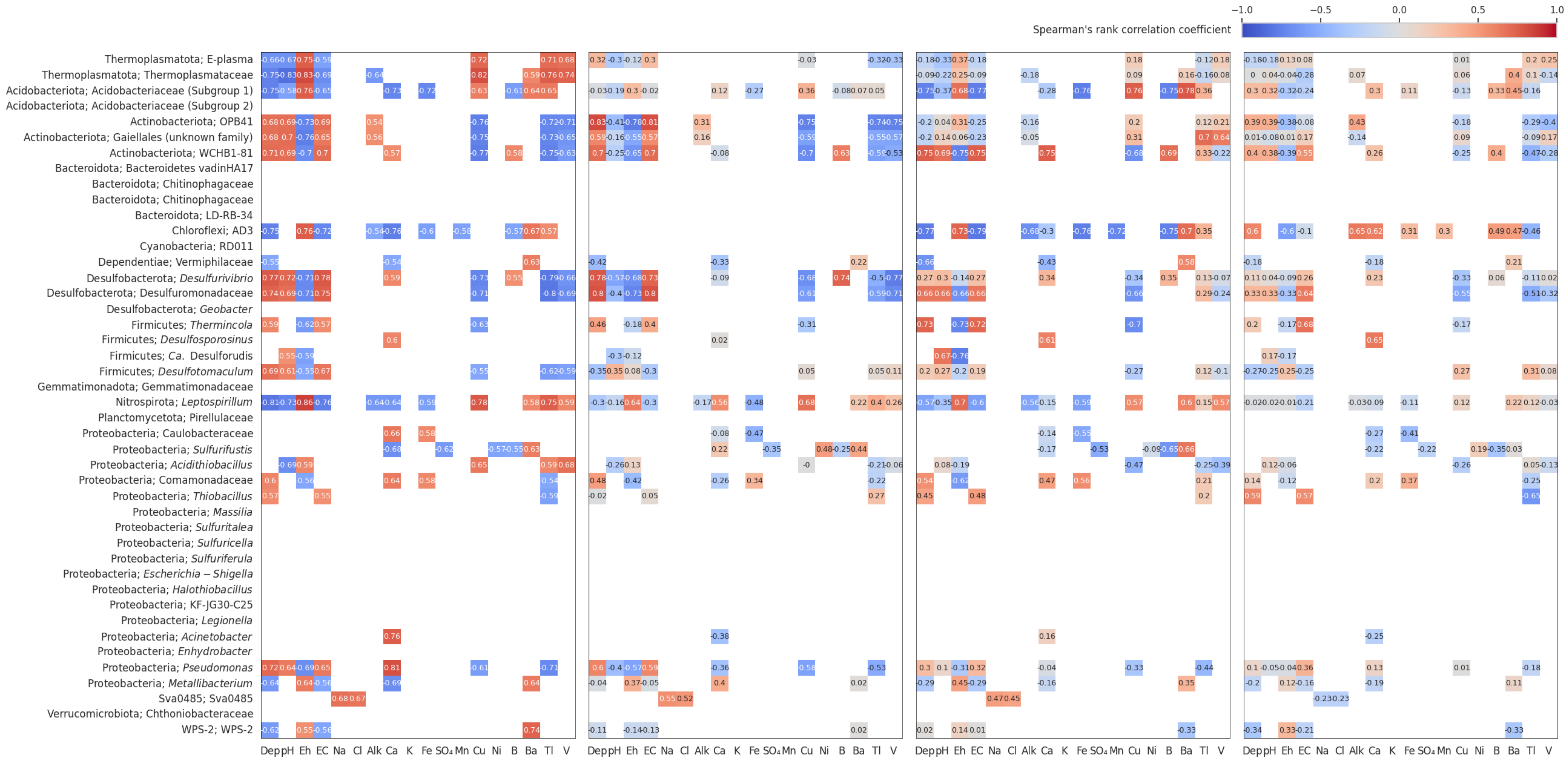

Supplemental Figure 2: Correlation matrices of genus-level relative abundance and geochemical factors (Dep = depth, EC = electrical conductivity, Alk = Alkalinity), subset by location. Spearman's rank correlation coefficients were calculated between the relative abundance of the genera present at >15% relative abundance in at least one sample (profiled in Figure 3) and select geochemical factors at each sampling location. Significant correlations ($p<0.05$, Bonferroni corrected) are shown.

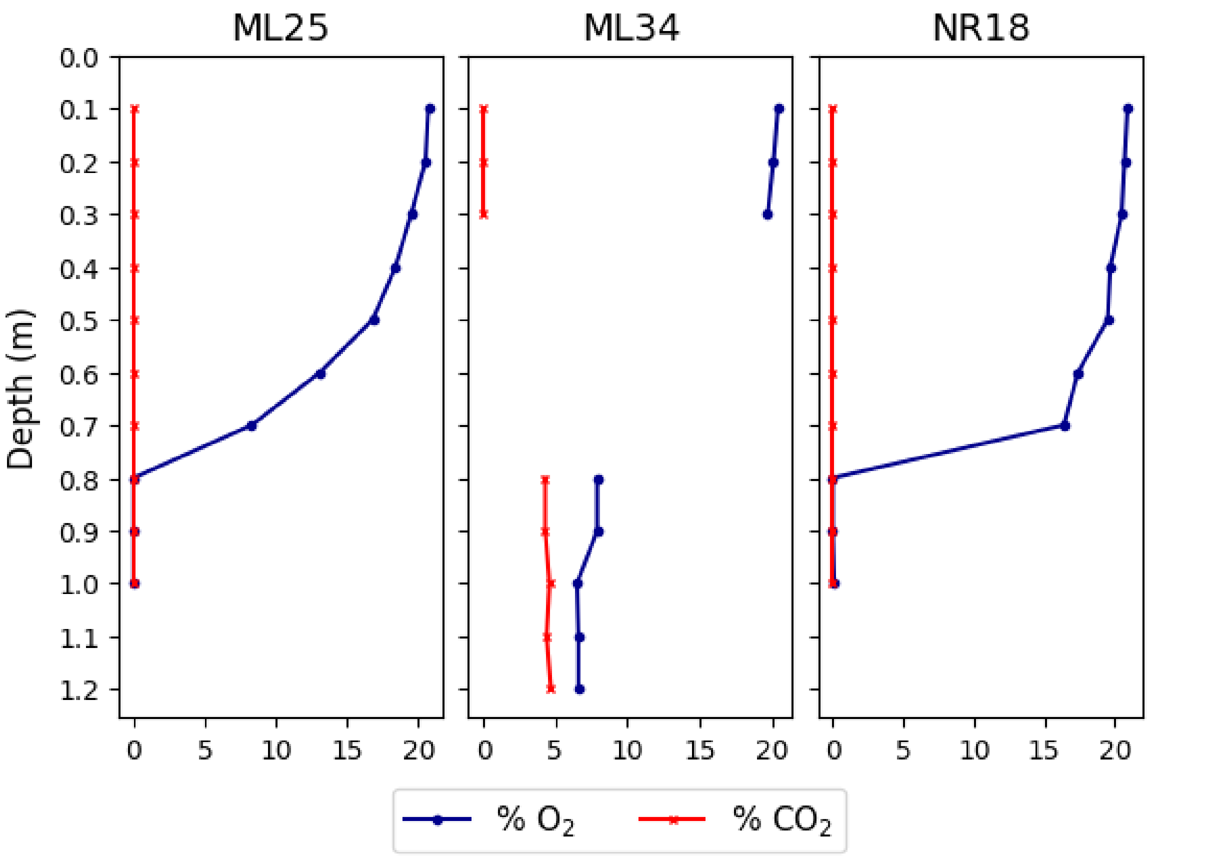

Supplemental Figure 3: Gas measurements of oxygen (% O_2) and carbon dioxide (% CO_2) in mine tailings. Oxygen decreased from atmospheric concentrations (20%) to 0.0% at ML25 and NR18 over the first 0.8 m. Depths below this point were saturated (below the water table). At ML34, depths between 0.4 – 0.8 m could not be measured. O_2 at ML34 dropped to 6.6% at the 1.2 depth, and further depths also could not be measured due to equipment malfunction and infiltration of atmospheric oxygen into the peristaltic pump. CO_2 was measured at 0.0% at all depth points at ML25 and NR18, while ML34 tailings showed CO_2 concentrations of 4.3 - 4.7% between 0.8 - 1.2 m, due to the dissolution of carbonate minerals present at this location.

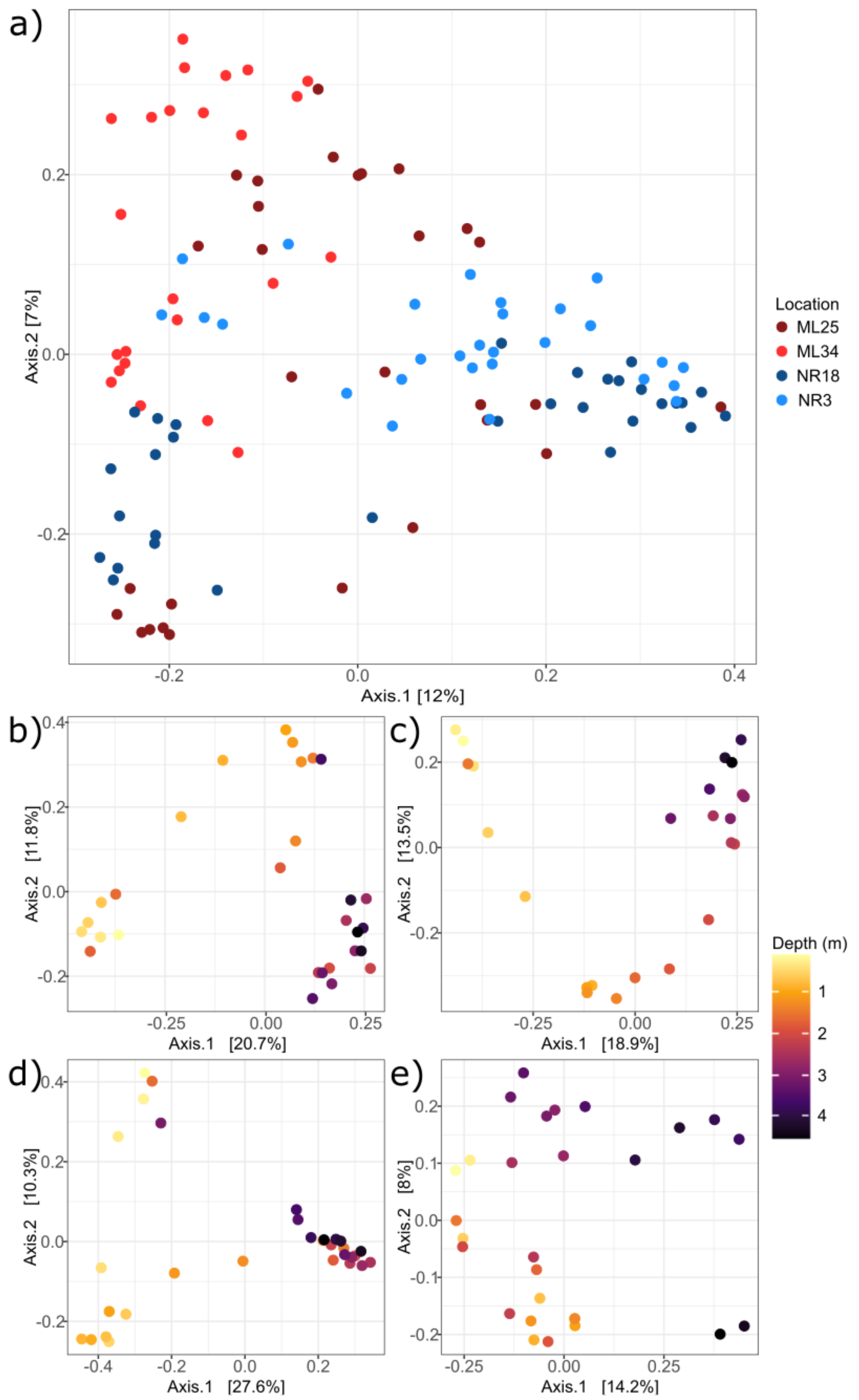

Supplemental Figure 4: Unweighted unifrac PCoA plot for a) all sampling locations combined, b) ML25, c) ML34, d) NR18, and e) NR3. Samples in plots b-e are colored by depth.

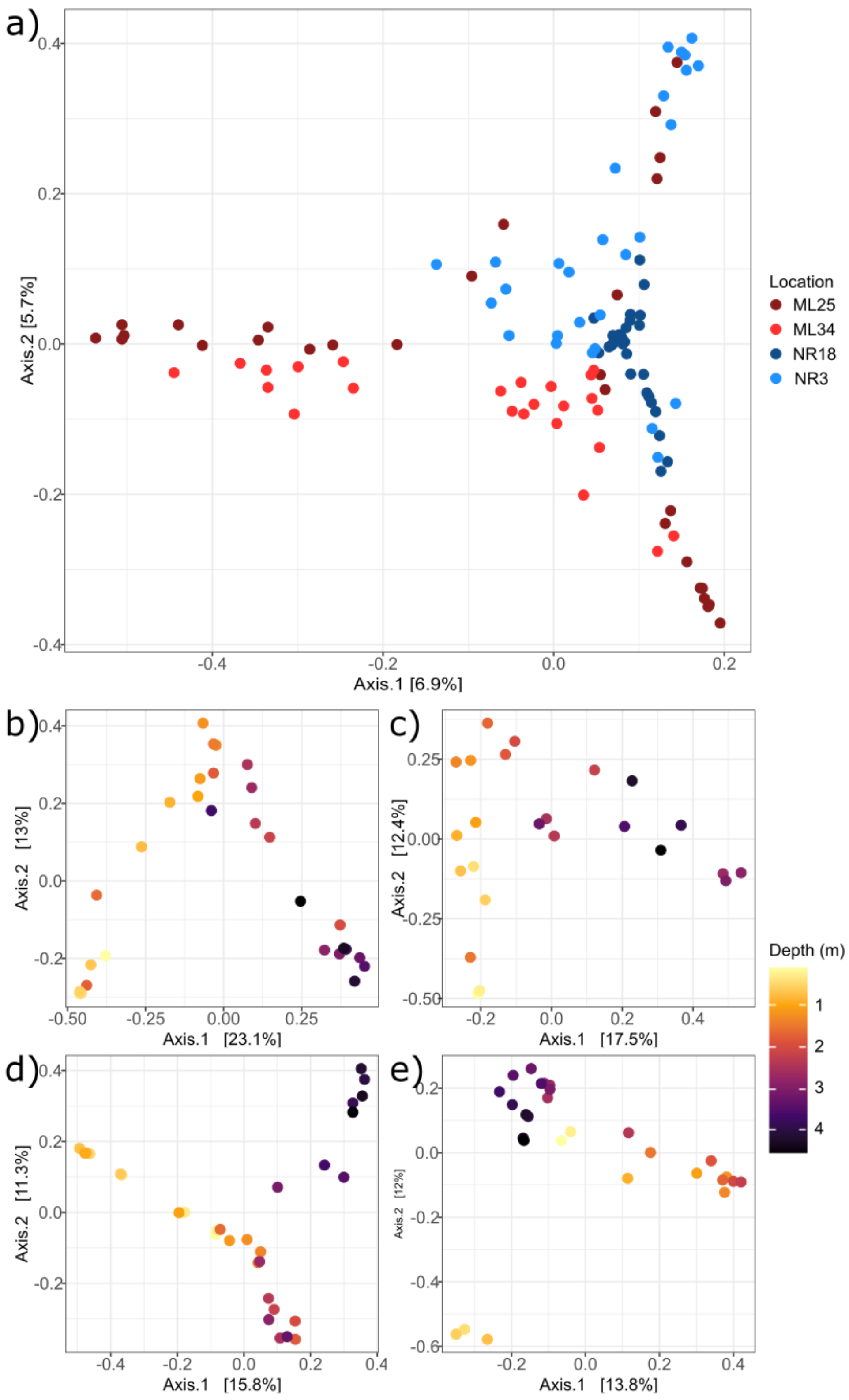

Supplemental Figure 5: Bray-Curtis PCoA plot for a) all sampling locations combined, b) ML25, c) ML34, d) NR18, and e) NR3. Samples in plots b-e are colored by depth.

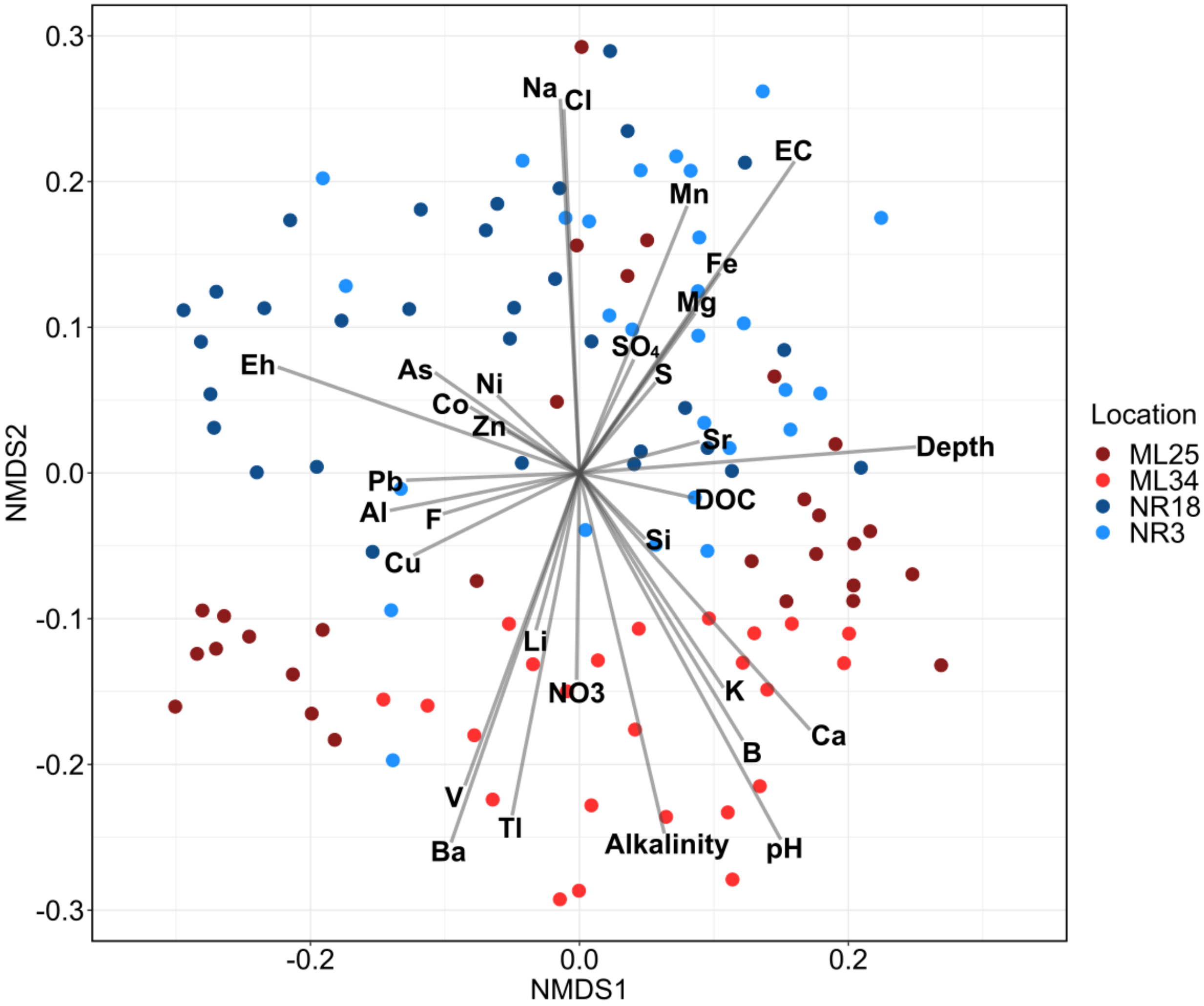

Supplemental Figure 6: NMDS of Bray-Curtis distances and environmental variables. All measured variables (with detectable values in >50% of samples, except for DOC and NO_3) are included. For raw data and p-values associated with geochemical variables, see Supplemental Data File 1.

Supplemental Table 1: Length and number of subsections of tailings cores by location. The length of each core was measured by the depth below ground level that was drilled to extract the core.

Location	Max depth below ground (m)	Number of subsections
**ML25**	4.66	29
**ML34**	4.60	24
**NR18 (2021)**	4.45	31
**NR18 (2019)**	4.75	31
**NR3**	3.90	28

Supplemental Data File 1: **Geochemistry measurements used for NMDS environmental fitting.** Data was measured from pore-water samples. Only factors which had measured values above the detection limit in >50% of samples in each location were included (with the exception of dissolved organic carbon (DOC) and NO_3, as they were expected to correlate with microbial diversity). Raw and adjusted (Bonferroni correction) p-values of environmental fitting vectors are shown in the bottom 2 rows; significant adjusted p-values (<0.05) are shaded in grey.
